# Experimental Evaluation of Performance in Polyethylene Terephthalate Modified Asphalt Mixtures Using Dry Mixing Methods

**DOI:** 10.3390/polym18050577

**Published:** 2026-02-27

**Authors:** Ba Tu Vu, Manh Tuan Nguyen

**Affiliations:** 1 Faculty of Civil Engineering, Ho Chi Minh City University of Technology (HCMUT), 268 Ly Thuong Kiet Street, Dien Hong Ward, Ho Chi Minh City 72500, Vietnam; 2 Vietnam National University Ho Chi Minh City, Linh Xuan Ward, Ho Chi Minh City 71300, Vietnam

**Keywords:** polyethylene terephthalate, polymer-modified asphalt, pavement materials, mixing method, fatigue life

## Abstract

High-quality pavement materials at reasonable prices are crucial for managing many heavy truck loads and hot weather conditions that present significant challenges for researchers, managers, and engineers. One effective strategy is to incorporate polymers into modified asphalt or asphalt mixtures. However, there are several notable challenges when using polymers in asphalt concrete, particularly related to mixing procedures and methods. Worldwide, two primary mixing methods are commonly used, including traditional dry and modified dry techniques. The dry method is usually preferred for using polyethylene terephthalate (PET) due to its various advantages. The indirect tensile strength, static resilient modulus, dynamic modulus, and fatigue tests were examined for all asphalt mixtures with PET using both dry methods. The findings from this research suggest that the modified dry mixing method is more effective, particularly regarding fatigue resistance, based on a systematic analysis of the results. In addition to these experimental investigations, an analysis of flexible pavement design for a typical pavement section has been conducted. This analysis utilized the experimental resilient modulus of all mixtures to predict fatigue life based on the Asphalt Institute model.

## 1. Introduction

Asphalt concrete, widely used in road construction, relies heavily on fossil-based materials, making it a costly and critical part of project budgets. Many road projects, whether involving new construction or maintenance, require large quantities of asphalt concrete, especially when high-quality pavement is essential. Polymer-modified asphalt pavement presents a potentially more suitable and cost-effective alternative for roadways, especially circular economic projects [1,2,3]. As society progresses, it also generates an increasing amount of plastic waste, including materials like polyethylene and polyethylene terephthalate (PET), which are difficult to decompose. Thermoplastics such as polyethylene and PET [3,4,5] can be utilized as waste materials in asphalt mixtures, benefiting both mechanical performance and the environment.

Many studies have indicated that incorporating PET as a partial replacement for aggregates or binder in asphalt mixtures can maintain or improve Marshall properties, fatigue life, moisture resistance, and rutting performance at low to moderate contents, while excessive PET replacement may reduce stiffness and fracture resistance [6,7,8,9,10,11,12,13]. Hassan et al. (2005) [6] utilized PET granules with an average diameter of approximately 3 mm as a 20% replacement for fine aggregate in mixtures. The results proved that both the Marshall stability and Marshall quotient of the PET-modified mixtures were nearly identical to those of the mixtures without PET. Nazmi and Wahab (2013) [7] evaluated the application of recycled PET particles, measuring 2 cm in size, as a partial replacement for fine aggregates in modified mixtures. Their study focused on permanent deformation and indirect tensile (IDT) stiffness, with PET contents ranging from 5% to 25% by weight of the asphalt mixture and fine aggregate sizes between 1.18 mm and 2.36 mm. The findings revealed that the maximum rutting occurred at a 20% replacement level of PET, while the stiffness of PET-modified mixtures generally decreased compared to unmodified mixtures. Hasan and Mohammad (2017) [8] examined the effects of waste PET at contents of 0%, 2%, 4%, 6%, 8%, and 10% by weight of binder on the engineering properties of asphalt concrete, including Marshall characteristics, IDT strength, moisture damage resistance, and dynamic creep performance. Two PET particle size ranges were used: coarse PET (1.18–2.36 mm) and fine PET (0.297–0.595 mm). The results demonstrated that the highest Marshall quotient was achieved at a 4% PET content for both particle sizes. In contrast, mixtures containing 2% PET exhibited the highest IDT strength values and greatest resistance to moisture damage. Moghaddam et al. (2012) [9] investigated the effects of incorporating waste PET with a maximum particle size of 2.36 mm into stone mastic asphalt (SMA) mixtures, focusing on stiffness and fatigue properties. PET contents ranged from 0.1% to 1% by weight of aggregates. The study revealed that PET-reinforced SMA mixtures exhibited significantly improved fatigue life, with an optimum PET content of 1% in terms of fatigue performance. Furthermore, Moghaddam et al. (2014) [10] reported that the permanent deformation resistance of asphalt mixtures was considerably enhanced through PET modification. Esfandabad et al. (2020) [11] evaluated the use of PET granules as a replacement for mineral aggregates in the size range of 2.36 mm to 4.75 mm, with replacement levels of 0%, 30%, 50%, 70%, and 100%. The study assessed IDT strength, moisture sensitivity, semi-circular bending, and wheel tracking performance. The results indicated that fracture resistance decreased when 30% and 50% PET replacement levels were applied in the asphalt mixtures. Laomuad et al. (2024) [12] improved the static and dynamic performance of RAP asphalt concrete by adding PET from 0 to 1.0% by weight of reclaimed aggregate. Suddeepong et al. (2024) [13] reported that PET at 0.2% to 0.6% enhanced the fatigue cracking and rutting resistance of asphalt concrete using reclaimed asphalt concrete and recycled concrete aggregate.

Studies on PET fiber-reinforced asphalt mixtures indicate that low to moderate PET fiber contents significantly enhance stiffness, cracking resistance, and rutting performance, whereas excessive fiber dosage leads to a reduction in stiffness [14,15]. In 2017, Usman et al. [14] studied the use of recycled PET fiber, which was 0.4 × 10 mm in size, at concentrations of 0.3%, 0.5%, 0.7%, and 1.0% by the weight of mixture. The results indicated that the stiffness modulus of PET-reinforced mixtures improved by 19%, 34%, and by 4% at 0.3%, 0.5%, and 0.7%, respectively. However, there was a decrease of 34% at 1.0% of recycled PET fiber compared to the control mixture. In a more recent study, Leiva-Villacorta and Cerdas-Murillo (2024) [15] utilized three different lengths of PET fibers, ranging from 57.1 mm to 63.5 mm, in hot mix asphalt and cold recycled mixture to evaluate their effects on cracking and rutting resistance. The results indicate that the PET fibers improved cracking resistance and positively influenced rutting performance, especially in hot mix asphalt.

The methods for mixing polymer-modified asphalt are essential for producing a consistent and high-quality product. Two well-known mixing methods are the dry process and the wet process [5,16,17,18,19,20,21]. In the dry process, polymer additives are pre-blended with dry aggregates before the asphalt binder is added. In contrast, the wet process involves mixing the polymer additives with the asphalt binder before combining them with the aggregates. However, the wet method is not suitable for certain plastics, such as PET, due to their high melting points (approximately 250 °C), which makes it challenging to achieve a uniform blend and increase the likelihood of separation from the asphalt binder [17]. Choudhary et al. [17] also noted that the modified dry mixing method, where PET is added after mixing the aggregates with asphalt, results in minimal changes to the shape and properties of PET during the mixing process. The dry mixing method is very convenient in Thailand production, especially for batching plants, where road construction contractors can directly incorporate plastic waste [18]. Haider and Hafeez (2021) [19] conducted a study on waste PET by adding different proportions of 3%, 6%, 9%, and 12% to asphalt cement to enhance its performance. They employed both wet and dry mixing methods at 160 °C. To evaluate the results, they utilized the tensile strength ratio, the Hamburg Wheel Track, and the Marshall immersion test. Their findings indicated that aggregates with a higher silica content were more sensitive to moisture. Lim et al. (2024) [20] investigated the feasibility of incorporating four common types of plastic, such as high-density polyethylene (HDPE), polypropylene (PP), polystyrene (PS), and PET, into asphalt concrete. They conducted several tests, including the Marshall stability, Cantabro abrasion, IDT strength, resilient modulus, Hamburg wheel tracking, and stripping test, to evaluate the mechanical properties of plastic-modified mixtures. This concrete contained different percentages of HDPE, PP, PS, and PET, and a dried mix approach was used. The results showed improvements in performance, although these improvements were less pronounced in the mixtures containing PS and PET.

All findings highlight the importance of selecting suitable PET forms, contents, and incorporation techniques. PET is primarily used in granules or fiber forms, with granules playing a more complex role, sometimes as an asphalt modifier and sometimes as an aggregate component of asphalt concrete mixtures. PET-modified asphalt can be produced using dry or wet mixing methods, but due to PET’s high melting point and its poor adhesion with asphalt binder, the dry process is generally more suitable and practical for PET incorporation for reasons including simplicity and compatibility with conventional asphalt plants, showing acceptable performance improvements despite being less effective than other plastics such as HDPE and PP. Despite these benefits, studies focusing on the performance of dry mixed PET-modified asphalt mixtures remain limited.

**Figure 1:**
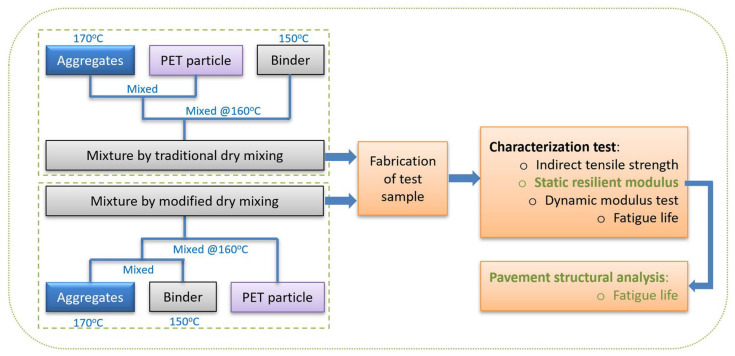
Flow chart of this study.

This study evaluates the feasibility and performance of asphalt concrete modified with recycled PET using both traditional and modified dry mixing methods, as illustrated in Figure 1. We systematically investigate the effects of incorporating PET on key mechanical properties, including indirect tensile strength, static resilience modulus, dynamic modulus, and fatigue resistance. The paper identifies the optimal PET content for both mixing methods, testing four levels of PET (0.2%, 0.4%, 0.6%, and 0.8% by weight of the aggregate) in comparison to a control mixture with 0% PET. This study aims to contribute to the development of sustainable materials and offer practical guidance for using recycled PET in asphalt pavement, particularly regarding fatigue resistance. Also, the structural response of the pavement was analyzed, emphasizing tensile strain or fatigue life and the effects of resilient modulus values from various mixtures.

## 2. Materials and Methods

This study evaluated the characteristics of asphalt mixtures that consist of recycled PET. Besides the control mixture, four types of PET-modified mixtures with PET content of 0.2%, 0.4%, 0.6%, and 0.8% by weight of the total aggregate and two dried mixing methods were selected for evaluation.

### 2.1. Materials

**Figure 2:**
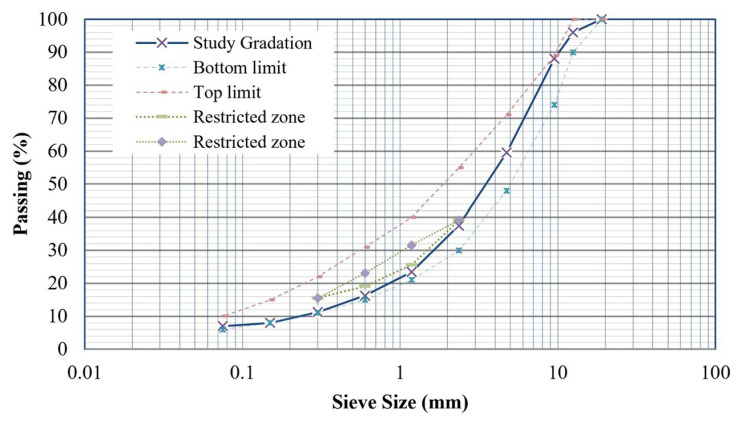
Chosen gradation in this study.

The aggregates utilized in this paper are crushed granite. A dense-graded material was selected, and this gradation adheres to the control limits specified in Table 1. Additionally, the selected gradation does not fall within a restricted zone, as indicated in Table 1 and Figure 2. The restricted zone is a specific area on the Superpave gradation chart [22], typically between 2.36 mm and 0.3 mm shown in Table 1, where the gradation should not pass through to prevent mixtures that are too fine and to avoid rutting performance. The aggregate properties are also presented in Table 2.

This study applied asphalt with a penetration grade of 60/70. The binder properties, including all of the main attributes of the asphalt binder, are shown in Table 3.

**Figure 3:**
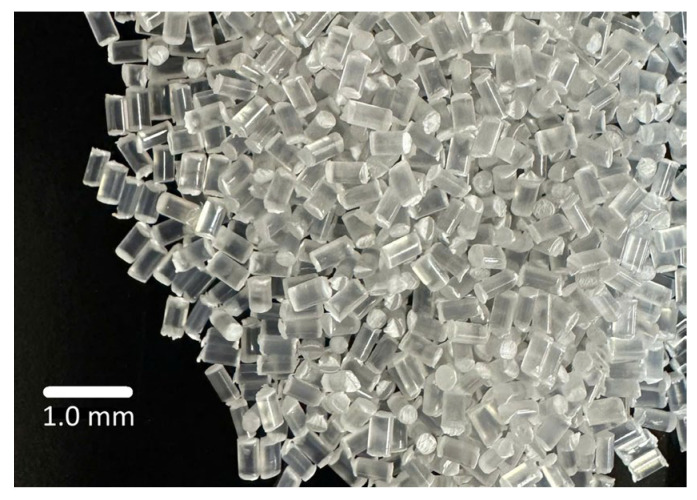
Recycled PET in this study.

This study focused on a specific type of recycled plastic known as polyethylene terephthalate (PET), which is visually represented in Figure 3. The recycled PET particles vary in size from 0.5 to 1.0 mm. The PET was obtained from a local company in Vietnam.

### 2.2. Mix Design

A mixture that did not contain any PET, termed control mixture, was utilized for the mix design. The optimal asphalt content was determined using the Marshall method [22] or TCVN 8820:2011 [23]. The following steps outline the methodology used for the mix design:

○ The chosen materials, including both aggregate and asphalt, have been carefully selected as outlined in Table 2 and Table 3. Additionally, the aggregate gradation is consistent with the specifications in Table 1.
○ The optimal asphalt content of mixture was determined by examining relationships among five asphalt contents and key properties, including bulk specific gravity, Marshall stability, flow, air voids (V_a_), voids in mineral aggregate (VMA), and voids filled with asphalt (VFA). The five chosen asphalt binder contents, increasing by 0.25 percent by weight of the mixture, were set at 4.5%, 4.75%, 5.0%, 5.25%, and 5.5%.

There are two methods outlined in TCVN 8820:2011 [23] for determining the optimal binder content. The first method establishes the optimal binder content based on achieving 4% air voids while ensuring that all properties meet the specifications for hot mix asphalt. The second method involves selecting the optimal binder content from a range where the key properties are all within acceptable limits.

**Figure 4:**
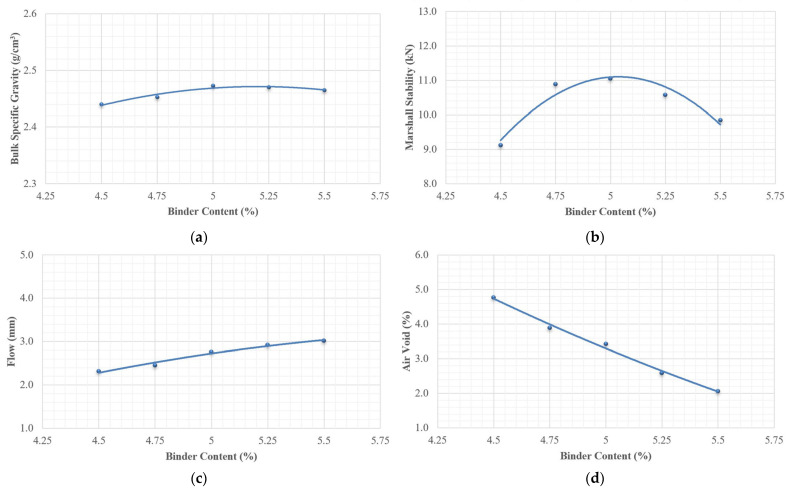

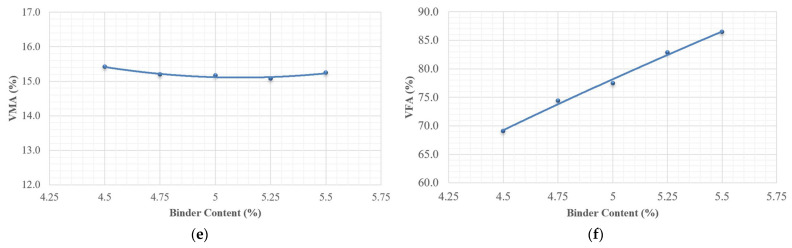
Relationships between binder content and asphalt mixture properties as follows: (**a**) bulk specific gravity; (**b**) Marshall stability; (**c**) flow; (**d**) air void; (**e**) VMA; (**f**) VFA.

Figure 4 compellingly demonstrates the relationships between binder content and several critical parameters: bulk specific gravity, Marshall stability, flow, V_a_, VMA, and VFA. According to the second method and the data presented in Figure 3, the optimal binder content is 5.0%. At this binder content, the measured values are as follows: air voids at 3.3%, Marshall stability at 11.0 kN, Marshall flow at 2.7 mm, VMA at 15.1%, and VFA at 78.1%.

### 2.3. Specimen Preparation

In our study, PET was added to the control mixture in amounts of 0.2%, 0.4%, 0.6%, and 0.8% by weight of the aggregate. The air void content in both the PET mixtures and the control mixtures were nearly identical, indicating that using PET does not compromise quality. We used two mixing methods: the traditional dry mixing method and the modified dry mixing method, as shown in Figure 1.

The modified dry mixing method involves the following steps: The asphalt and aggregates are heated at 150 °C for 2 h and at 170 °C for more than 4 h, respectively. After heating, the asphalt and aggregates are mixed at 160 °C, and then the PET particles are directly put into this mixture.

In contrast, the traditional dry mixing method first combines the aggregates and PET. After that, the asphalt binder was added to this mixture at 160 °C.

Three types of specimens were prepared for this study as follows:

(1) Cylindrical asphalt concrete specimens were created using a Matest Marshall compactor. Typical specimens have a diameter of 101.6 mm and a height of approximately 63.5 mm. These specimens were utilized for indirect tension strength and fatigue tests.

(2) Cylindrical asphalt concrete specimens were carefully prepared using a hydraulic compressor at a pressure of 30 MPa. Each specimen has the same diameter and height of 100 mm. These specimens were subsequently utilized for conducting static resilient tests.

(3) Cylindrical specimens have the same diameter and height of 150 mm and were prepared using a hydraulic compressor at 30 MPa. Then, the cored specimens, which have a diameter of 100 mm and a height of 150 mm, were drilled from the cylindrical specimens. The core specimens were used for dynamic modulus tests.

### 2.4. Characterization

#### 2.4.1. Indirect Tensile Strength Test

IDT strength was determined following the procedure specified in AASHTO T 283 [24]. The samples were first conditioned at 25 °C in a controlled environment for at least 2 h to ensure uniform temperature distribution. A vertical load was then applied diametrically to each specimen at a constant displacement rate of 50 mm/min until fracture. The peak load at failure (P) was recorded and subsequently used to compute the IDT strength using the following equation:

(1) $$S_{t}=\frac{2P}{\pi \mathrm{HD}}$$

where

P = applied max load (N);

H = specimen thickness or height (mm);

D = specimen diameter (mm).

#### 2.4.2. Static Resilient Test

Before testing, the specimens were stored in a controlled environment at 15 °C, 30 °C, and 60 °C, in compliance with Russian and Vietnamese specifications [25], to ensure thermal equilibrium. We applied a constant pressure (p) of 0.5 MPa on the specimen to determine the static resilient modulus. The measured resilience (L) indicates the difference in sample displacement along the thickness with and without the applied pressure (p). The static resilient modulus (E) was then defined using the following expression:

(2) $$E=\frac{\mathrm{pH}}{L}$$

where

p = loading pressure (MPa);

H = specimen thickness or specimen height (mm);

L = resilient value (mm).

#### 2.4.3. Dynamic Modulus Test

The dynamic modulus test was conducted based on AASHTO TP62 [26]. A servo-hydraulic material testing system with a 30 kN load cell and a temperature-controlled chamber was employed to apply cyclic axial loading. Two LVDTs installed symmetrically on both sides of the specimen were used to record axial deformation. Measurements were conducted at five temperatures (−10, 4, 21, 37, and 54 °C) and six loading frequencies (25, 10, 5, 1, 0.5, and 0.1 Hz).

The dynamic modulus |E*| in MPa is calculated as follows:

(3) $$\left|E*\right|=\frac{\sigma_{o}}{\varepsilon_{o}}$$

where

σ_o_ = the peak value of axial loading stress (MPa);

ε_o_ = the recoverable axial strain.

#### 2.4.4. Fatigue Test

IDT fatigue testing was performed in stress-controlled mode at 20 °C in accordance with EN 12697-24:2012 [27]. Specimens were subjected to repeated haversine compressive loading at 10 Hz until fracture. The horizontal tensile stress and strain were derived using Equations (4) and (5), and the stiffness was obtained from the stress–strain ratio. Fatigue life was determined as the cycle number corresponding to a 50% reduction in initial stiffness. All asphalt mixtures were evaluated under stress levels of 150 kPa, 250 kPa, and 350 kPa.

(4) $$\sigma =\frac{2F}{\pi \mathrm{HD}}$$

(5) $$\varepsilon =\frac{2\left(\frac{\Delta H}{D}\right)\left(1+3\upsilon \right)}{\left(4+\pi \upsilon -\pi \right)}$$

where

F = applied force (N);

D and H are the specimen diameter and height (mm);

ΔH = the horizontal deformation (mm);

ν = 0.35 is Poisson’s ratio.

### 2.5. Structural Analysis

The resilient modulus values of nine asphalt mixtures at 15 °C were utilized to assess the influence of static resilient modulus on fatigue life. These values were obtained from static resilient modulus tests for mixtures and were utilized for pavement structure analysis using the KENLAYER program [28].

A typical pavement section, detailed in Table 4, was analyzed to evaluate the effect of static resilient modulus on pavement performance, specifically regarding fatigue life. In this analysis, the thicknesses of both the asphalt layer and the base layer were kept constant across all cases. Poisson’s ratio was 0.35 for the asphalt layer and graded aggregate base, and 0.45 for the subgrade. The mechanistic analysis also employed a contact pressure of 600 kPa and a contact radius of 165 mm.

## 3. Results and Discussion

The conclusions drawn from the experiment are outlined below. Furthermore, the analysis of flexible pavement design for a typical pavement section based on the experimental resilient modulus of nine mixtures has been established to predict fatigue lives.

### 3.1. Evaluation Results from Two Mixing Methods

#### 3.1.1. Indirect Tensile Strength

**Figure 5:**
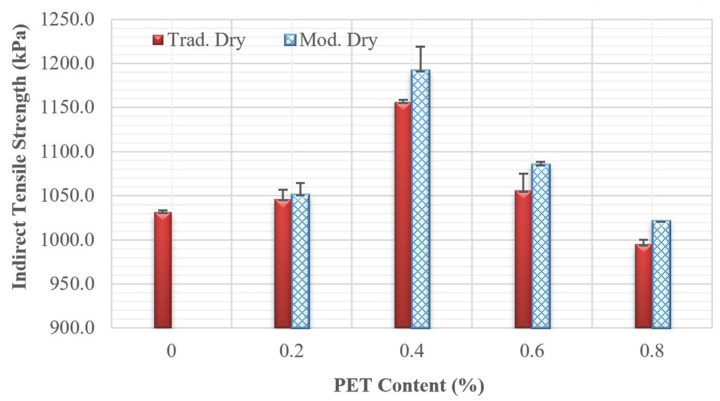
Tensile strength of all mixtures.

Figure 5 illustrates that PET enhances cracking resistance, particularly as the PET content increases up to 0.6% compared with the mixture without PET. The results indicate that the asphalt concrete mixture achieves its highest tensile strength at a PET content of 0.4% for both mixing methods. Notably, the modified dry mixing method outperformed the traditional dry mixing method, especially at the 0.4% PET content, where the modified method demonstrated an improvement of approximately 3.1% over the traditional method. However, when the PET content increases to 0.8%, the IDT strength of the mixture decreases, falling below the strength observed when no PET is used. Since PET does not melt under conventional mixing temperatures, it remains as discrete solid particles. At higher dosages, particle agglomeration becomes more likely, leading to non-uniform dispersion and the formation of weak zones.

#### 3.1.2. Static Resilient Modulus

**Figure 6:**
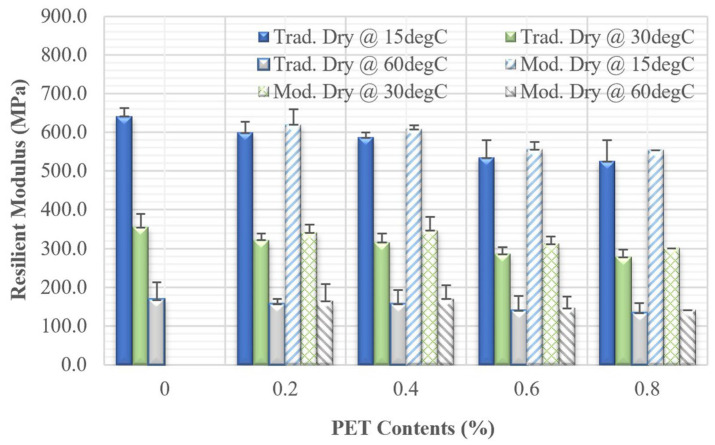
Resilient modulus of all mixtures.

The static resilient modulus of the nine asphalt mixtures measured at 15 °C, 30 °C, and 60 °C is presented in Figure 6. It can be observed that higher PET content results in lower resilient modulus values at all temperatures. For example, at 15 °C, using the traditional dry mixing method, the elastic modulus of asphalt concrete with PET at contents of 0.2%, 0.4%, 0.6%, and 0.8% decreased by 6.5%, 8.5%, 16.6%, and 18.1%, respectively, compared to asphalt concrete without PET. Similarly, at 15 °C, using the modified dry mixing method, the resilient modulus of mixture containing PET at the same content levels showed reductions of 3.4%, 5.2%, 13.2%, and 13.5%, respectively, compared to the asphalt concrete without PET. The trend observed at the experimental temperatures of 30 °C and 60 °C followed a similar pattern to that seen at 15 °C, highlighting consistent effects between the two mixing methods. For all three temperature tests, the modulus from the modified dry mixing is higher than that from the traditional dry mixing method.

#### 3.1.3. Dynamic Modulus

**Figure 7:**
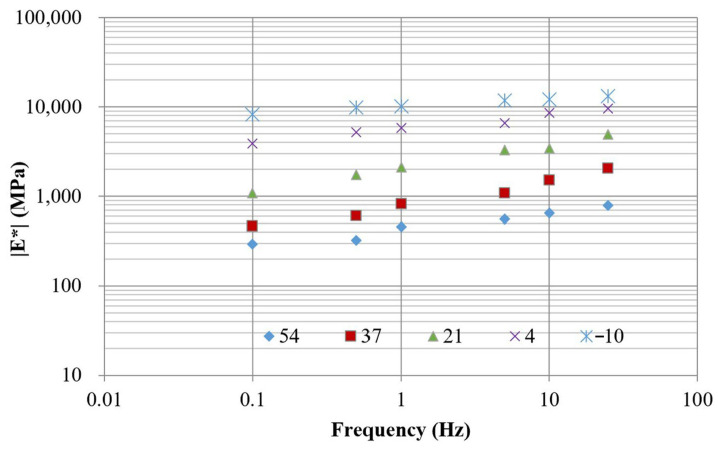
Dynamic modulus of mixture without PET.

Figure 7 illustrates the dynamic modulus results of the control mixture (without PET) obtained at temperatures of −10 °C, 4 °C, 21 °C, 37 °C, and 54 °C under loading frequencies of 25, 10, 5, 1, 0.5, and 0.1 Hz. The modulus data at different temperatures were shifted along the frequency axis until they merged into a single sigmoidal curve, referred to as the master curve. The time–temperature superposition principle was applied to construct the master curve [29], which aligns data collected at various temperatures to generate a continuous and smooth representation of material behavior. Typically, the master curve can be mathematically represented by

(6) $$\log\left|E^{*}\right|=\delta +\frac{\alpha }{1+e^{\beta +\gamma \left({\mathrm{logf}}_{r}\right)}}$$

where

f_r_ = reduced frequency of loading at reference temperature;

δ = minimum value of E*;

δ + α = maximum value of E*;

β, γ = parameters describing the shape of sigmoidal function.

The shift factor is as follows:

(7) $$a(T)=\frac{f_{r}}{f}$$

where

a(T) = shift factor as a function of temperature;

f = frequency at desired temperature;

f_r_ = reduced frequency at reference temperature;

T = temperature of interest.

**Figure 8:**
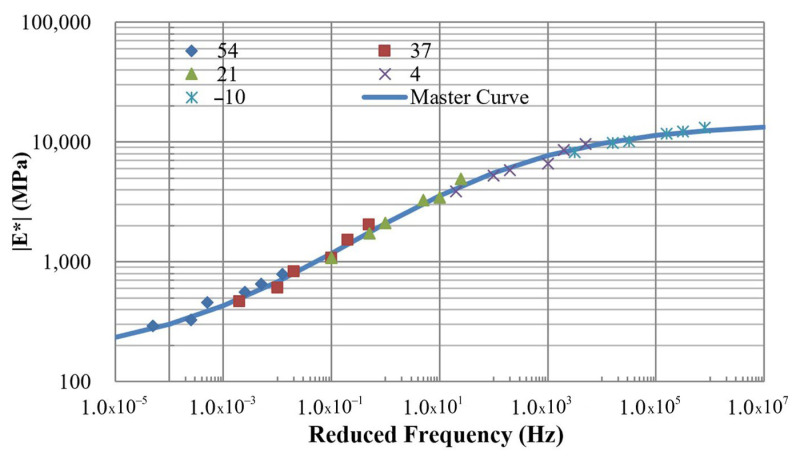
Master curve for the mixture without PET at a reference temperature of 21 °C.

**Figure 9:**
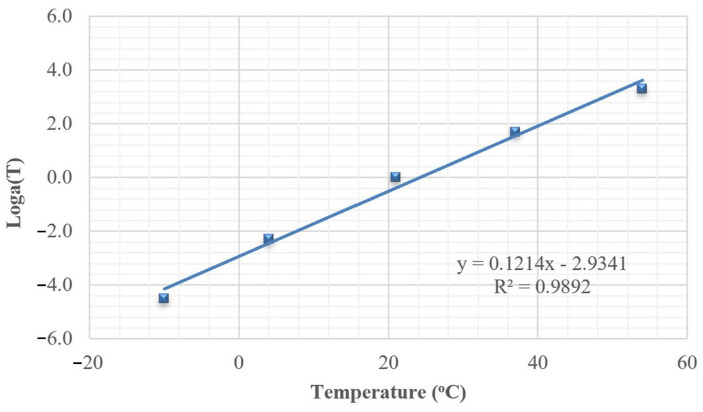
Shift factor versus temperature for mixture without PET.

The modulus master curve, expressed in terms of reduced frequency, describes the viscoelastic response of the material over time. A reference temperature of 21 °C was selected for this analysis. The frequency shift applied at different temperatures to construct the master curve (Figure 8) was governed by the shift factor. This factor was calculated from the regression analysis shown in Figure 9, which exhibits a strong correlation with an R^2^ value of 0.9892. For the mixture without PET, the master curve is represented by four parameters of a sigmoidal function, described in Equation (6): δ, α, β, and γ, which are 4.17, −2, 0.3, and 0.5, respectively.

**Figure 10:**
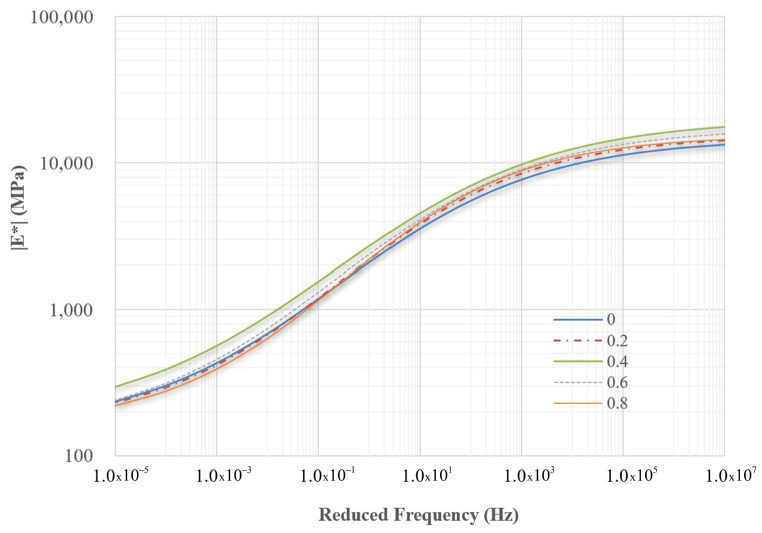
The master curves for mixtures using the traditional dry mixing method.

**Figure 11:**
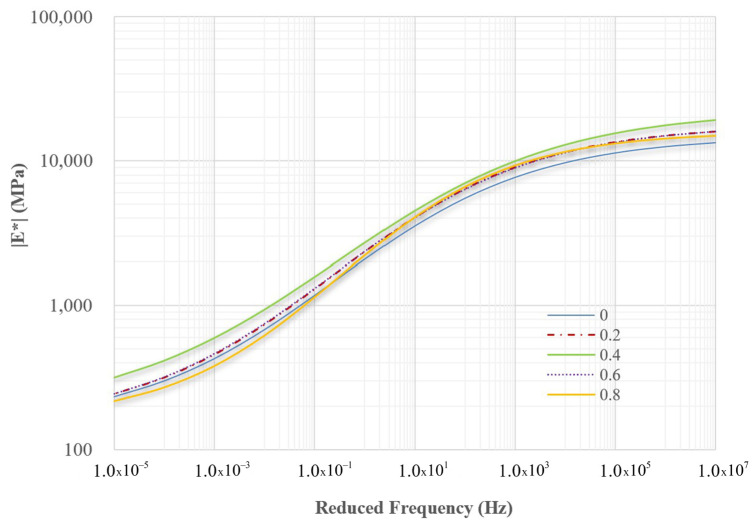
The master curves for mixtures using the modified dry mixing method.

Master curves for the other mixtures were obtained from experimental dynamic modulus data and are displayed in Figure 10 and Figure 11. For comparison, the mixture without PET, referred to as the control mixture, is also included in these figures. The four constants of the sigmoidal function for all nine mixtures are listed in Table 5, based on regression analysis.

**Figure 12:**
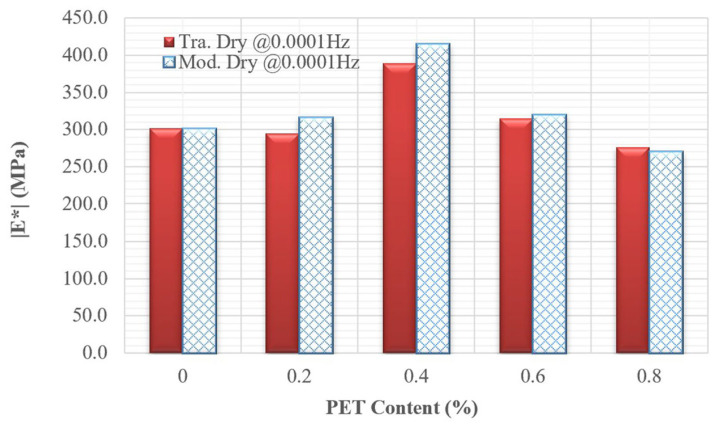
Bar chart of dynamic modulus for nine mixtures at low reduced frequency.

**Figure 13:**
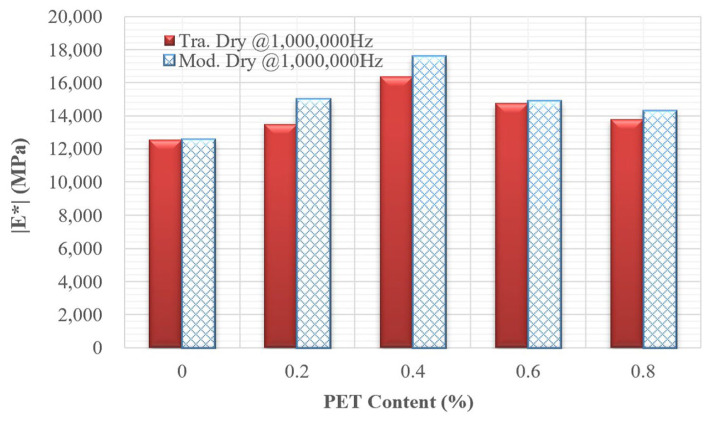
Bar chart of dynamic modulus for nine mixtures at high reduced frequency.

Two reduced frequencies, 0.0001 Hz and 1,000,000 Hz, were selected to evaluate the dynamic modulus values of all mixtures. Low frequencies are associated with materials that operate at hot temperatures or under slow loading rates, while high frequencies correspond to materials functioning at low temperatures or under rapid loading rates. Figure 12 and Figure 13 illustrate the dynamic modulus values at these low and high frequencies for nine different mixtures. At low frequencies, PET improved the workability of asphalt concrete by 0.6%. However, when the concentration of PET reached 0.8%, the workability decreased compared to mixtures without PET. In contrast, at high frequencies, PET consistently enhanced the quality of the asphalt concrete compared to mixtures that did not contain PET. Regardless of whether the mixtures were evaluated at low or high frequencies, the mixture containing 0.4% PET exhibited the best overall quality. Furthermore, all dynamic modulus values indicate that the quality of modified dry mixing is superior to that of traditional dry mixing.

#### 3.1.4. Fatigue Life

The fatigue life was evaluated based on the criterion that considers half of the stiffness. All mixtures were subjected to testing at three constant stress levels: 150 kPa, 200 kPa, and 250 kPa. The fatigue lives of mixtures at three stress levels are summarized in Table 6.

**Figure 14:**
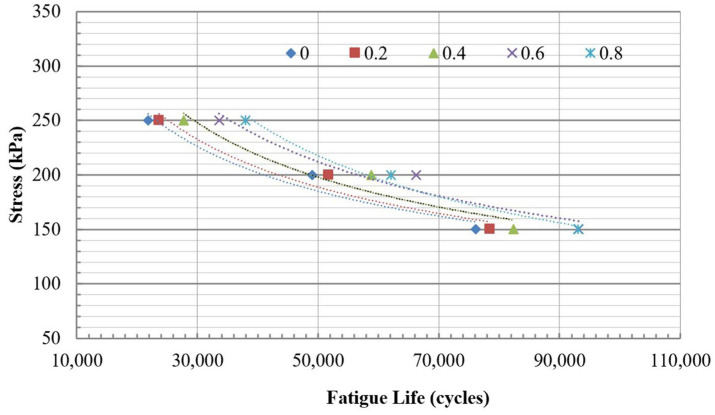
The relationship between fatigue lives and stress levels for mixtures using traditional dry mixing.

**Figure 15:**
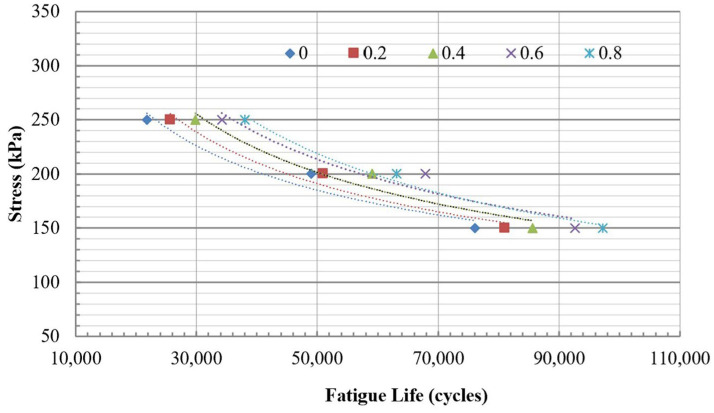
The relationship between fatigue lives and stress levels for mixtures using modified dry mixing.

The fatigue lives were presented in correlation with three different stress levels, as shown in Figure 14 and Figure 15 for both the traditional and modified dry mixing methods. The relationship between fatigue lives and stress levels can be expressed using a power regression model, as illustrated in formula (8). Regression analysis reveals this relationship, and the results are summarized in Table 7. The R^2^ presented in Table 7 indicates that these relationships are robust, with values exceeding 0.9.

(8) $$N_{f}=k_{1}{\left(\sigma \right)}^{k_{2}}$$

where

N_f_ = fatigue life of mixture (cycles);

σ = stress level (kPa);

k_1_ and k_2_ = model coefficients provided in Table 7.

**Table 7:** Statistical analysis results for the fatigue model in Equation (8).

Mixing Type	PET Content (%)	Model Coefficients		Coefficient of Determination R^2^
		k_1_	k_2_	
Without PET (Control mixture)	0	12,950.27	−0.393	0.94
Traditional dry mixing	0.2	15,678.93	−0.408	0.94
	0.4	23,345.34	−0.441	0.92
	0.6	36,783.03	−0.477	0.93
	0.8	99,464.69	−0.566	0.98
Modified dry mixing	0.2	21,324.93	−0.436	0.97
	0.4	30,712.66	−0.465	0.94
	0.6	39,238.94	−0.482	0.92
	0.8	76,060.66	−0.541	0.99

Figure 14 and Figure 15 illustrate that the addition of PET significantly improves the fatigue life of mixtures, regardless of whether traditional or modified dry mixing methods are utilized. According to Table 6, at a stress level of 200 kPa, the fatigue life of mixtures containing 0.2%, 0.4%, 0.6%, and 0.8% PET using the traditional dry mixing method increased by 3.2%, 8.2%, 22.5%, and 22.4%, respectively, compared to the mixture without PET. In the same way, at a stress level of 200 kPa, the fatigue life of mixtures with 0.2%, 0.4%, 0.6%, and 0.8% PET using the improved dry mixing method increased by 6.4%, 12.5%, 21.8%, and 27.7%, respectively, compared to the mixture without PET. In most cases, the modified dry mixing method provides slightly better fatigue resistance than the traditional dry method, depending on the levels of PET and stress applied.

### 3.2. Structural Analysis in Fatigue Lives

The key structural responses measured in flexible pavement include the tensile strain at the bottom of the asphalt layer and the vertical compression strain at the top of the subgrade [22,28]. To address these issues, the Asphalt Institute in the United States developed a mechanical method for designing flexible pavements based on the two key criteria mentioned above.

Using KENLAYER, the results of the pavement’s structural response were analyzed, focusing on tensile strain as shown in Table 8, which details the effects of nine resilient modulus values from various mixtures. The results indicated that the highest tensile strain occurred in the mixture without PET. As the PET content increased from 0.2% to 0.4%, 0.6%, and 0.8%, the tensile strain progressively decreased. This trend can be attributed to the fact that the mixture without PET exhibited the highest elastic modulus value compared to the other mixtures, leading to the greatest strain value. Moreover, the modified dry mixing method leads to slightly lower tensile strain compared to the traditional dry mixing method.

In this article, the fatigue resistance of the asphalt mixture was evaluated by using the Asphalt Institute (AI) model to predict the fatigue life [22]. The relationship between the fatigue life of asphalt concrete and the tensile strain at the bottom of the asphalt concrete layer can be expressed as follows:

(9) $$N_{f}={0.0795\varepsilon }_{t}^{-3.291}E^{-0.854}$$

where

N_f_ = Fatigue life of asphalt concrete (cycles).

ε_t_ = Tensile strain at the bottom of asphalt concrete layer.

E = Resilient modulus of asphalt concrete (psi); this resilient modulus is influenced by temperature and applied load levels. The fatigue life is defined as the point when fatigue cracking covers 10% of the asphalt concrete pavement’s surface area.

**Figure 16:**
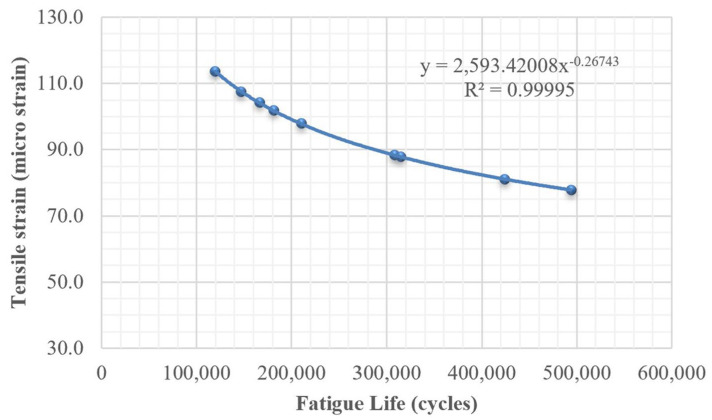
The relationship between fatigue life and tensile strains of nine mixtures.

The fatigue performance of the nine mixtures was estimated using the AI model, and the predicted values are reported in Table 7. Overall, the incorporation of PET resulted in improved fatigue resistance when compared with mixtures without PET, and a progressive enhancement in performance was observed as the PET content increased. Moreover, a clear distinction between the two mixing techniques was identified, with the modified dry mixing method yielding lower fatigue resistance than the traditional dry mixing method. By combining the tensile strain (ε_t_) and fatigue life (N_f_) results of all nine mixtures in Figure 16, a power-type relationship between tensile strain and fatigue life was derived, exhibiting an excellent fit with an R^2^ value of 0.99995. The fatigue life and tensile strain can be expressed using the following regression equation:

(10) $$N_{f}=2593.42\varepsilon_{t}^{-0.27}$$

There is the same power form in Equations (9) and (10) for fatigue life prediction in fatigue test and fatigue analysis from an AI model. The difference between Equations (9) and (10) is the tensile stress and tensile strain.

In summary, experimental results and analysis of flexible pavement structures indicate that employing high PET content can enhance the fatigue resistance of asphalt concrete mixtures with thin layers. Based on these experimental results, the modified dry mixing method was chosen for application. Additionally, AI model analysis supports the selection of the traditional dry mixing method due to fatigue performance.

## 4. Conclusions

This study focuses on evaluating the quality of PET-modified asphalt concrete mixtures using two dry mixing methods: traditional dry mixing and modified dry mixing. Firstly, the hot mix asphalt without PET or the control mixture was designed using the Marshall method. Then, four PET contents of 0.2%, 0.4%, 0.6%, and 0.8% were added to the control mixture to form the PET-modified asphalt concrete mixture. Two dry mixing methods were used for evaluation in laboratory tests, including indirect tensile testing, static resilient modulus, dynamic modulus, and indirect tensile fatigue testing. Mechanistic pavement analysis was conducted to evaluate the tensile strain at the bottom of the asphalt concrete layer for different static resilient modulus values, and fatigue life was predicted using the Asphalt Institute model. The following conclusions are derived from the analysis of laboratory test results and pavement structure modeling:

○ The IDT strength improved with the addition of PET up to 0.6%, but it decreased when the PET content increased to 0.8%. The modified dry mixing method produced better tensile strength compared to the traditional method. Both methods indicated that the optimal PET content for achieving the highest indirect tensile strength was 0.4%.
○ Increasing the content of PET reduces the static resilient modulus across all three temperature conditions.
○ Based on the dynamic modulus values, the addition of PET improved the workability of asphalt concrete until 0.6%. However, when the content of PET reached 0.8%, the workability decreased compared to mixtures without PET, especially in low-frequency or high-temperature conditions. Regardless of whether the mixtures were evaluated at low or high frequencies, the mixture containing 0.4% PET exhibited the best overall quality. The quality of modified dry mixing is superior to that of traditional dry mixing in terms of dynamic modulus.
○ PET addition significantly enhances the fatigue life of asphalt concrete mixtures. Moreover, the improved dry mixing method provides slightly better fatigue resistance than the traditional dry mixing method based on fatigue testing.
○ Based on the mechanistic analysis for a typical asphalt pavement section, all fatigue lives based on the Asphalt Institute model indicated that asphalt concrete mixtures containing PET exhibited better fatigue resistance than those without PET. The fatigue resistance of the modified dry mixing method was lower than that of the traditional dry mixing method.

In general, PET addition enhanced the cracking resistance of asphalt concrete mixtures based on indirect tensile strength, dynamic modulus, and fatigue life from both experimental and analytical evaluations. The results demonstrated that PET additives contribute positively to pavement performance. Although the modified dry mixing method showed longer fatigue life in experimental tests, the analytical analysis showed that the traditional method performed better between the two mixing methods.

Furthermore, 1176 tons of asphalt concrete are needed for a 1 km section, with a width of 8 m and a 6 cm asphalt surface thickness. If an amount of 0.4 to 0.6% PET is used, the amount of PET needed for that road would be 4.47 to 6.70 tons or approximately 223,500 to 335,000 500 mL PET bottles. For each 500 mL PET bottle recycled, 0.53 MJ of energy is saved compared to the landfill solution [30]. As a result, the 1 km road can save between 118.455 MJ and 177.550 MJ when using PET-modified asphalt concrete.

## Figures and Tables

**Table 1:** Aggregate gradation.

Sieve Size (mm)	Percent Passing				
	Chosen Gradation	Control Points		Restricted Zone Boundary	
		Low Limit	High Limit	Minimum	Maximum
19.0	100.0	100.0	100.0		
12.5	96.0	90.0	100.0		
9.5	88.0	75.0	89.0		
4.75	59.5	48.0	71.0		
2.36	37.5	30.0	55.0	39.1	39.1
1.18	23.4	21.0	40.0	25.6	31.6
0.6	16.25	15.0	31.0	19.1	23.1
0.3	11.25	11.0	22.0	15.5	15.5
0.15	8.0	8.0	15.0		
0.075	7.0	6.0	10.0		

**Table 2:** Aggregate properties.

Type	Properties	Result
Coarse aggregate	Specific gravity, g/cm^3^	2.70
	Los Angeles abrasion, %	14.64
	Elongation ratio, %	10.73
	Adhesive with binder	Level 4
Fine aggregate	Specific gravity, g/cm^3^	2.59
	Sand equivalent, %	83
Mineral filler	Specific gravity, g/cm^3^	3.00

**Table 3:** Basic binder properties.

Properties	Result
Penetration @25 °C, 0.1 mm	63
Softening point, °C	48
Ductility @25 °C, cm	152
Aggregate adhesive with binder	Level 4
Specific gravity, g/cm^3^	1.03
Flash point, °C	>350

**Table 4:** The pavement section in this study.

Layer	Thickness (cm)	Poisson’s Ratio	Resilient Modulus at 15 °C (MPa)
Asphalt concrete	6	0.35	Varying based on nine mixture types, as shown in Figure 6 or Table 8
Base (Graded Aggregate)	30	0.35	300
Subgrade	Infinite	0.45	47

**Table 5:** The constants of the sigmoidal function for the master curves of nine different mixtures.

Mixing Type	PET Content (%)	δ	α	β	γ
Without PET (Control mixture)	0	4.17	−2	0.3	0.5
Traditional dry mixing	0.2	4.19	−2	0.3	0.53
	0.4	4.3	−2.07	0.32	0.47
	0.6	4.24	−2.07	0.33	0.5
	0.8	4.19	−2	0.3	0.56
Modified dry mixing	0.2	4.25	−2.07	0.3	0.5
	0.4	4.35	−2.1	0.25	0.45
	0.6	4.25	−2.08	0.31	0.49
	0.8	4.2	−2	0.29	0.58

**Table 6:** Fatigue lives of nine mixtures.

Mixing Type	PET Content (%)	Stress (kPa)		
		150	200	250
Without PET (Control mixture)	0	76,097	49,010	21,833
Traditional dry mixing	0.2	78,503	51,706	23,689
	0.4	82,362	58,833	27,766
	0.6	93,188	66,206	33,598
	0.8	93,108	62,060	38,000
Modified dry mixing	0.2	81,002	50,978	25,689
	0.4	85,619	58,999	29,800
	0.6	92,675	67,890	34,210
	0.8	97,201	63,100	37,987

**Table 8:** The pavement analysis results from nine mixtures.

Mixing Type	PET Content (%)	Resilient Modulus (kPa)	Tensile Strain (Micro Strain)	Fatigue Life (Cycles) from AI Model
Without PET (Control mixture)	0	641,644	113.6	119,489
Traditional dry mixing	0.2	599,967	101.8	181,547
	0.4	586,987	97.9	210,487
	0.6	534,852	81.1	423,851
	0.8	525,270	77.7	493,957
Modified dry mixing	0.2	620,167	107.6	147,066
	0.4	608,417	104.2	166,149
	0.6	556,852	88.4	307,873
	0.8	555,370	87.9	314,271

## Data Availability

The data presented in this study are available on request from the corresponding author.
